# Long-Term Outcomes of Bariatric Surgery: A Systematic Review

**DOI:** 10.7759/cureus.39638

**Published:** 2023-05-29

**Authors:** Murtajiz M Raza, Temiloluwa Njideaka-Kevin, Jordan Polo, Khawaja Azimuddin

**Affiliations:** 1 Department of Research, Avalon University School of Medicine, Willemstad, CUW; 2 Department of Surgery, Houston Methodist Willowbrook Hospital, Houston, USA

**Keywords:** depression, roux-en-y gastric bypass, gastric bypass, cancer, cardiovascular diseases, morbidly obese, bariatric surgery

## Abstract

Roux-en-Y gastric bypass (RYGB) is a minimally invasive procedure that usually takes two hours. It is commonly performed in refractory cases to help morbidly obese patients (BMI ≥ 40 kg/m2) lose weight. It is well known that morbid obesity accompanies other comorbid conditions such as atherosclerotic diseases, strokes, cancers, and mental health issues such as anxiety and depression. It is crucial to treat this category of patients to improve their quality of life and minimize the chance of mortality in these patients. Given the importance of treating this group, we explored the long-term outcomes of patients who underwent bariatric surgery for cardiovascular diseases, cancer, and depression compared to those who did not. This systematic review utilized articles identified through PubMed using the following search terms: (morbidly obese OR obesity OR obese) AND (bariatric surgery OR metabolic surgery OR gastric bypass OR gastrectomy) AND (chronic disease OR chronic diseases OR cardiovascular diseases OR heart diseases OR cancer OR neoplasms OR stroke OR depressive disorder OR depression). The filter used was observational studies, which yielded 217 studies. Out of these results, eight citations were included in an observational study that met our eligibility criteria. From our search, the articles showed a clinically significant decrease in the incidence of cardiovascular disease, cancer, and depressive disorders after treatment with bariatric surgery. Furthermore, there was also a correlation between bariatric surgery and remission of type 2 diabetes. The surgery has an apparent protective effect on the development and progression of comorbid conditions accompanying morbid obesity. Overall, the quality of life has improved in patients who have undergone the procedure compared to those who have not. Bariatric surgery must be recommended as a beneficial option in managing morbidly obese patients (BMI ≥ 40 kg/m2) who have responded poorly to first-line management plans.

## Introduction and background

Morbid obesity is a growing problem in the United States as obesity rates continue to rise yearly, not only in the United States but worldwide [1,2]. It is strongly associated with an increased risk of several adverse health effects, including cardiovascular diseases, cancer, and depression. Previous studies have shown that obese patients are approximately four times more likely to have hypertension and increased cancer incidence. Cancer is due to converting the high-fat diet supplied with fatty acids, or de novo synthesized fatty acids, into protumorigenic signaling lipids incorporated in oncogenic signaling lipids that drive cancer pathogenicity [3]. Morbidly obese patients are also more likely to suffer from multisystem problems such as orthopedic problems and obstructive sleep apnea that adversely impact the quality of their daily life, leading to mental health conditions such as anxiety and depression [4]. As healthcare professionals, we strive to effectively manage the problems that impair our patients' optimal functioning and health.

Early stages of obesity, if not addressed appropriately, can result in progression to morbid obesity and thereby increase its prevalence [1,5]. Many forms of nonsurgical therapy to manage morbid obesity are dietary modifications and daily exercises. Still, it is hard to tell how efficiently these methods will decrease the prevalence of morbid obesity. We believe the assistance of another type of therapy, along with the abovementioned methods, will help patients drastically lose weight.

One of the many treatments for morbid obesity is bariatric surgery, which aims to decrease caloric intake and stimulate weight loss in morbidly obese patients by creating a gastric bypass or sleeve gastrectomy. This procedure has successfully transformed patients' body figures and overall health [1-5].

Our hypothesis is if patients with morbid obesity (BMI ≥ 40 kg/m2) undergo bariatric surgery, then there will be a lower rate of incidence of cardiovascular diseases, cancer, and depression compared with a matched cohort of patients with morbid obesity (BMI ≥ 40 kg/m2) who did not receive the intervention of bariatric surgery [1,3,4]. We intend to dissect this topic and analyze the qualities of evidence by conducting a systematic review study design to summarize the results of several primary articles.

## Review

Methods

Search Strategy

We searched the PubMed database exclusively for observational studies from 2014 to 2022. We combined the following search terms from four themes/concepts: morbidly obese (BMI ≥ 40 kg/m2), bariatric surgery, no bariatric surgery, cardiovascular diseases, cancer, and depression. The terms were searched as Medical Subject Heading (MeSH), free words, keywords, or synonyms using the Boolean operators OR and AND. The search strategy was as follows: (morbidly obese OR obese OR overweight) AND (bariatric surgery OR gastric bypass OR gastrectomy OR gastric sleeve surgery) AND (no bariatric surgery) AND (cardiovascular disease OR strokes OR cardiovascular accidents OR myocardial infarction OR cancer OR depression). The filter used was observational studies. The total number of citations we identified through our search strategy was 217. The retrieved citations were then exported to an Excel spreadsheet (Microsoft Corporation, Redmond, WA) and screened by title and abstracts. We excluded citations based on the initial screening of the title and abstracts if they did not relate to our research hypothesis/objective and were not original studies, editorial/case reports, or commentary articles. Disagreements were resolved by consensus.

Study Selection and Eligibility Criteria

**Figure 1:**
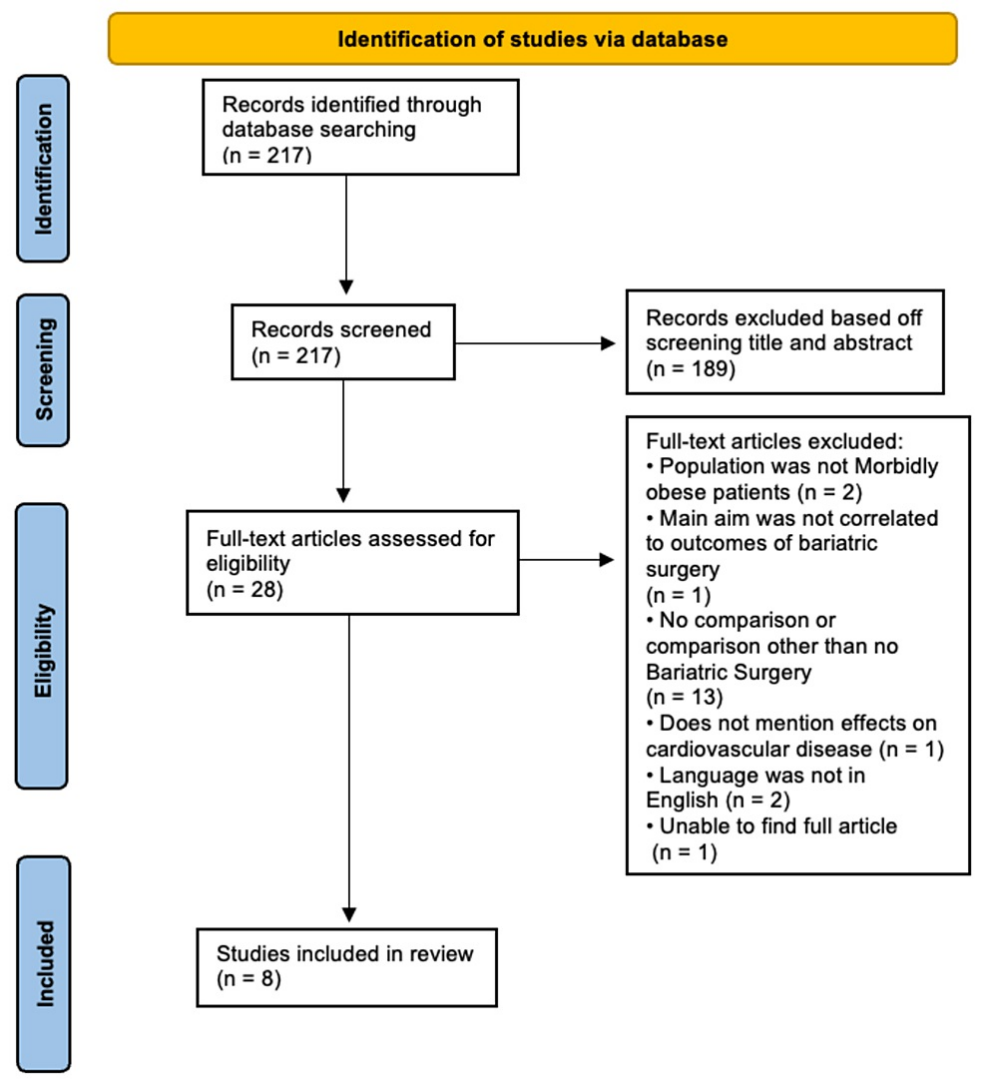
PRISMA flowchart PRISMA: Preferred Reporting Items for Systematic Reviews and Meta-Analyses.

Our patient/population, intervention, comparison, and outcomes (PICO) concepts screened full-text articles of the citations that met the abstract and title screening. To be included in this review, the full-text screening should mention the following: (i) morbidly obese (BMI ≥ 40 kg/m2); (ii) bariatric surgery; (iii) no bariatric surgery; and (iv) cardiovascular diseases, cancer, and depression. We excluded articles if they used the following: (i) population other than morbidly obese (BMI ≥ 40 kg/m2); (ii) interventions other than bariatric surgery; (iii) comparisons other than no bariatric surgery; (iv) outcomes other than cardiovascular diseases, cancer, and depression; and (v) study designs other than observational studies (Table 1). Our study selection is presented in Figure 1.

**Table 1:** Inclusion and exclusion criteria

Inclusion criteria	Exclusion criteria
1. Population of morbidly obese patients with BMI ≥40 kg/m2	1. Population not limited to morbidly obese patients with BMI ≥ 40 kg/m2
2. Bariatric surgery	2. Main aim was not correlated to the outcomes of bariatric surgery
3. No bariatric surgery	3. Comparisons other than bariatric surgery are not the focus
4. Cardiovascular diseases	4. Does not mention the effects on cardiovascular disease
5. Cancer	5. Does not mention the effects on cancer
6. Depression	6. Does not mention the effects of depression
7. Observational studies	7. Study was not an observational study
	8. Not an original study/editorial/case report/commentary guideline articles/reports
	9. Retracted studies

Measurement and Observation: Outcome, Exposures, and Covariates

The exposures were morbidly obese patients with a body mass index (BMI) ≥ 40 receiving bariatric surgery. The outcomes were about decreasing a patient's risk of cardiovascular disease, heart disease, depression, depressive disorder, cancer, neoplasms, stroke, and chronic illness in the future.

Assessment of Methodological Quality

The included studies were assessed using the Newcastle-Ottawa Quality Assessment Scale (NOS) [6]. Each study was assessed to determine if it satisfied the following criteria: (i) the non-exposed and exposed cohort was selected from the same community; (ii) the individuals in the studies were representative of the average target population; (iii) the exposure was ascertained through secure records or structured interview; (iv) the study demonstrated that the outcome(s) of interest was not present at the start of the study; (v) the study adjusted for more than one confounding; (vi) the study assessed the outcome(s) through an independent blind assessment or record linkage; (vii) the follow-up period was long enough for the outcome(s) of interest to occur; (viii) the loss to follow-up (i.e., the percentage of individuals in the selected population sample who actively participated in the study, but did not complete the study) was 10% or less. Each quality item was assessed with a "yes" if adequate data were provided or clearly and adequately described vital characteristics and a "no" if the item criteria were not met. Items were classified as "no description" if the study provided no available data. Disagreements in any quality assessment items for each study were resolved by consensus (Table 2).

**Table 2:** Quality indicators of included studies

Study	Study sites	Reported on lost to follow-up	Ascertainment of exposure	Assessment of outcome	Adjusted for confounders	Outcomes defined
Schauer et al. (2019) [1]	USA	No	Secure records	Record linkage	Yes	Yes
Inge et al. (2016) [2]	Not specified	Yes	Structured interview	Independent blind study	No	Yes
Aravani et al. (2018) [3]	Sweden and England	Yes	Secure records	Record linkage	Yes	Yes
Gordon et al. (2019) [4]	Not specified	Yes	Secure records	Self-report	Yes	Yes
Persson et al. (2017) [5]	Sweden	Yes	Secure records	Self-report	Yes	Yes
Lent et al. (2017) [7]	Not specified	No	Secure records	Hospital records	Yes	Yes
Eliasson et al. (2015) [8]	Sweden	Yes	Secure records	Self-report	Yes	Yes
Lundby-Christensen et al. (2014) [9]	Denmark	No	Secure records	Record linkage	Yes	Yes

Data Extraction

The following data were extracted from each study: sample size, sex, race, study type, mean BMI, study sites, number of cases (cancer, cardiovascular events, or depression), and loss to follow-up. Discrepancies during the data extraction process were resolved by consensus.

Data Analysis

The articles were analyzed by investigating the results of the exciting outcomes of each piece. The results of similar outcomes were then compared between articles.

Results

Description of Studies

The computer database search yielded 217 citations for assessment. The initial screening of titles and abstracts identified 28 studies that potentially met the inclusion criteria. After the texts were reviewed in total, 20 citations were excluded. The remaining eight studies were included in our longitudinal cohort (Figure 1).

The eight studies assessed a total of 1,251,113 individuals. The studies were executed in various countries: two in Sweden, one in Sweden and England, one in Denmark, and one in the USA. The last three studies did not state their locations. The male-to-female ratio of the studies ranged from 1:4.9 to 1:1.4 (Table 3).

**Table 3:** Characteristics of studies included in the prospective longitudinal cohort for long-term outcomes of bariatric surgery

Study	Country	Number of patients in the study	Male (%)	Female (%)	Cardiovascular symptoms (%)	Cancer symptoms (%)	Depression symptoms (%)	BMI, kg/m^2^
Schauer et al. (2019) [1]	USA	88,625	18.88%	81.1%	60%	99%	–	≥44.6
Inge et al. (2016) [2]	–	88,625	25%	75%	43%	–	–	≥44.6
Aravani et al. (2018) [3]	Sweden and England	1,002,607	36.5%	63.5%	–	1.05%	–	≥40
Gordon et al. (2019) [4]	–	6,215	21.3%	78.7%	68%	–	–	≥40
Persson et al. (2017) [5]	Sweden	47,859	–	–	33.25%	2.9%	–	≥40
Lent et al. (2017) [7]	–	4,856	16.9%	83.1%	87%	–	53%	≥45
Eliasson et al. (2015) [8]	Sweden	12,264	39%	61%	17.2%	–	–	≥40
Lundby-Christensen et al. (2014) [9]	Denmark	62	42%	58%				≥40

Of the eight studies included, four had a retrospective cohort study design, and four had a prospective longitudinal cohort study design. Most studies ascertained the exposure through secure hospital records, with only one study utilizing a structured interview to confirm the exposure to bariatric surgery. All the studies defined their outcomes; only one did not adjust for confounding. Only six studies reported the loss of follow-up. Only two studies focused on more than one outcome of interest; however, no study focused on all outcomes (Table 2).

Study Outcomes

The study outcomes included a decreased risk of cardiovascular disease (including stroke and myocardial infarctions), reduced risk of depressive disorder, and decreased risk of cancers, as illustrated in Table 4.

**Table 4:** Main results CI: confidence interval; CRC: colorectal cancer; SIR: significant increase risk; OS: obesity surgery; BDI-1: Beck Depression Inventory-1; HR: hazard ratio; RYGB: Roux-en-Y gastric bypass; IMT: intima-media thickness; T2D: type 2 diabetes; IGT: impaired glucose tolerance; NGT: normal glucose tolerance.

Study	Study design	Main findings
Schauer et al. (2019) [1]	Retrospective cohort study	Patients undergoing bariatric surgery had a 33% lower hazard of developing any cancer during follow-up (hazard ratio = 0.67, 95% CI = 0.60, 0.74, p < 0.001).
Inge et al. (2016) [2]	Retrospective cohort study	Three years after the procedure, remission of type 2 diabetes occurred in 95% (95% CI = 85 to 100) of participants who had had the condition at baseline, remission of elevated blood pressure in 74% (95% CI = 64 to 84), and remission of dyslipidemia in 66% (95% CI = 57 to 74).
Aravani et al. (2018) [3]	Retrospective cohort study	In the no-surgery obese population, 3,237 developed CRC (SIR 1.12 (95% CI = 1.08-1.16)). Of those who underwent OS, 43 developed CRC (SIR 1.26 (95% CI = 0.92-1.71)). The OS cohort demonstrated decreased breast cancer risk (SIR 0.76 (95% CI = 0.62-0.92)), unlike the no-surgery cohort (SIR 1.08 (95% CI = 1.04-1.11)). Increased endometrial and kidney cancer risk was observed in surgery and no-surgery cohorts.
Gordon et al. (2019) [4]	Prospective longitudinal cohort study	The prevalence of self-harm/suicidal ideation (BDI-1) was 5.3% (95% CI = 3.7-6.8) presurgery and 3.8% (95% CI = 2.5-5.1) at year one postsurgery (p = 0.06). Prevalence increased over time postsurgery to 6.6% (95% CI = 4.6-8.6) at year 5 (p = 0.001) but was not significantly different than presurgery (p = 0.12).
Persson et al. (2017) [5]	Prospective longitudinal cohort study	Patients who underwent bariatric surgery had a markedly reduced risk of heart failure compared with nonsurgical obese patients (age- and sex-adjusted hazard ratio (HR) = 0.37, 95% CI = 0.29-0.46). The lower risk persisted after further adjustment for baseline differences in known risk factors for heart failure (HR = 0.37, 95% CI = 0.30-0.46).
Lent et al. (2017) [7]	Retrospective cohort study	Overall, RYGB patients experienced a significant reduction in the risk of all-cause mortality compared with control subjects (HR = 0.65 (95% CI = 0.50, 0.84); p < 0.0001). 58 patients and 43 controls were taking antidepressant medications.
Eliasson et al. (2015) [8]	Prospective longitudinal cohort study	We noted a 58% relative risk reduction (HR = 0.42, 95% CI = 0.30-0.57; p < 0.0001) in overall mortality in the RYGB group compared with the controls. The risk of fatal or non-fatal myocardial infarction was 49% lower (HR = 0.51, 95% CI = 0.29-0.91; p = 0.021), and that of cardiovascular death was 59% lower (0.41, 0.19-0.90; p = 0.026) in the RYGB group than in the control group. Five-year absolute risks of death were 1.8% (95% CI = 1.5-2.2) in the RYGB group and 5.8% (5.0-6.8) in the control group.
Lundby-Christensen et al. (2014) [9]	Prospective longitudinal cohort study	Mean carotid IMT was significantly reduced 12 months after RYGB in patients with T2D/IGT (-0.041 mm (95% CI = -0.069, -0.012, p = 0.005)) but not in patients with NGT (-0.010 mm (-0.039, 0.020, p = 0.52)).

After follow-up by five articles, the depression outcomes show the prevalence of suicidal ideation was 5.3% (95% CI = 3.7-6.8) presurgery [4] and 3.8% (95% CI = 2.5-5.1) at year one postsurgery (p = 0.06) [4]. Prevalence increased over time postsurgery to 6.6% (95% CI = 4.6-8.6) at year five (p = 0.001) but was not significantly different from pre-surgery (p = 0.12), although patients still had a decrease in reported postoperative depression symptoms [3,4]. In the study by Lent et al. (2017), however, the use of antidepressants among patients undergoing Roux-en-Y gastric bypass (RYGB) with diabetes was 53% compared to controls with diabetes, which had 46% (Table 3) [7]. The non-diabetic counterparts undergoing the RYGB procedure had a 55% use of antidepressants [7]. The postoperative RYGB patients without diabetes were likelier to die from intentional self-harm than similar patients who did not undergo the RYGB procedure [7].

As an outcome, two studies reported the risk of developing cancers after exposure to bariatric surgery. Schauer et al. (2019) calculated a hazard ratio of 0.67 (95% CI = 0.60-0.74) for all cancers [1]. Aravani et al. (2018) compared the risk of two cancer types between the bariatric surgery and the control group [3]. For colorectal cancer, the group that underwent bariatric surgery had a standardized incidence ratio (SIR) of 1.26 (95% CI = 0.92-1.71), while the control group had a SIR of 1.12 (95% CI = 1.08-1.16). The bariatric surgery group for breast cancer had a SIR of 0.76 (95% CI = 0.62-0.92), while the control group had a SIR of 1.08 (95% CI = 1.04-1.11).

The cardiovascular outcomes revealed a significant decrease in cardiovascular adverse effects. In a study by Inge et al. (2016), patients with bariatric surgery showed a reduction in elevated blood pressure by 74% (95% CI = 64-84) [2] and dyslipidemias by 66% (95% CI = 57-74) [2]. Persson et al. (2017) showed that heart failure was reduced compared to the control group (age- and sex-adjusted HR = 0.37, 95% CI = 0.29-0.46) [5]. In a study by Eliasson et al. (2015), myocardial infarction was reduced by 49% (HR = 0.51, 0.29-0.91; p = 0.021) [8]. Lundby-Christensen et al. (2014) mentioned that the mean carotid intima-media thickness (IMT) was significantly reduced 12 months after RYGB in patients with type 2 diabetes (-0.041 mm (95% CI = -0.069, -0.012, p = 0.005)) (Table 4) [9].

Discussion

Morbid obesity is rising and is a significant risk factor for ischemic heart disease, the leading cause of death worldwide. In this systematic review, we focused on bariatric surgery as a treatment option for morbid obesity and its ability to modify the potential incidence of three key long-term health outcomes: cardiovascular disease, cancers, and depressive disorders.

After assessing the results of the included articles, all articles showed clinically significant decreased incidence of cardiovascular disease with a 63% relative risk reduction, cancers with a 33% relative risk reduction, and depressive disorders after treatment of morbid obesity with bariatric surgery. There was also a correlation between bariatric surgery and remission of type 2 diabetes, hypertension, and hyperlipidemia (Table 4).

Overall, mental health crises are still a concern whether patients undergo bariatric surgery. However, bariatric surgery and the proper care and management, like psychotherapy and antidepressants, can drastically improve how patients feel about themselves when they see weight loss effects from the surgery. They will have a better sense of self-esteem and self-image, which will help their mental health, but the probability of self-harm should not be neglected [7].

Scrutinizing the quality of each study showed an overall high quality. All included studies clearly defined their outcomes, and all studies from two ascertained their products through hospital records. Only one study did not state how the exposure was established. However, three studies did not report on loss to follow-up, and as a result, these critical quality criteria needed to be satisfied.

Strengths include using studies with patients who received bariatric bypass surgery and comparing the outcomes with control groups without treatment. This study also included studies in the US, Sweden, and Denmark. The methodological quality of the included studies was rigorously assessed using the NOS.

The weaknesses of this study could be attributed to the small sample size and the use of only observational studies. Some articles contained only some of the specific outcomes we were looking for. Along with that, there were few articles about cancer and depression. We also did not look at articles that were randomized clinical trials or clinical trials, which limited us to the level of evidence and clinical recommendations.

## Conclusions

In conclusion, bariatric surgery has an apparent protective effect on the development of significant diseases in morbidly obese patients. With laparoscopic surgeries becoming more common than laparotomies, RYGB is done through minimally invasive keyhole-sized incisions, causing a decrease in scar formation that is more cosmetically appealing for patients. Also, laparoscopic procedures only need a few hours to perform, have fast recoveries, and generally have no significant postoperative complications. This procedure can assist morbidly obese patients who struggle with diets and exercise regimens to lower their BMI and their chance of mortality. Patients that are morbidly obese and healthcare practitioners should be aware of this treatment option to improve the patient's quality of life.
